# Prevalence and associated factors of internet addiction among undergraduate university students in Ethiopia: a community university-based cross-sectional study

**DOI:** 10.1186/s40359-020-00508-z

**Published:** 2021-01-06

**Authors:** Yosef Zenebe, Kunuya Kunno, Meseret Mekonnen, Ajebush Bewuket, Mengesha Birkie, Mogesie Necho, Muhammed Seid, Million Tsegaw, Baye Akele

**Affiliations:** 1 Department of Psychiatry, College of Medicine and Health Sciences, Wollo University, Dessie, Ethiopia; 2 Department of Pharmacy, College of Medicine and Health Sciences, Wollo University, Dessie, Ethiopia

**Keywords:** Internet addiction, Students, Ethiopia

## Abstract

**Background:**

Internet addiction is a common problem in university students and negatively affects cognitive functioning, leads to poor academic performance and engagement in hazardous activities, and may lead to anxiety and stress. Behavioral addictions operate on a modified principle of the classic addiction model. The problem is not well investigated in Ethiopia. So the present study aimed to assess the prevalence of internet addiction and associated factors among university students in Ethiopia.

**Objectives:**

Main objective of this study was to assess the prevalence and associated factors of internet addiction among University Students in Ethiopia.

**Methods:**

A community-based cross-sectional study was conducted among Wollo University students from April 10 to May 10, 2019. A total of 603 students were participated in the study using a structured questionnaire. A multistage cluster sampling technique was used to recruit study participants. A binary logistic regression method was used to explore associated factors for internet addiction and variables with a *p* value < 0.25 in the bivariate analysis were fitted to the multi-variable logistic regression analysis. The strength of association between internet addiction and associated factors was assessed with odds ratio, 95% CI and *p* value < 0.05 in the final model was considered significant.

**Results:**

The prevalence of internet addiction (IA) among the current internet users was 85% (n = 466). Spending more time on the internet (adjusted odds ratio (AOR) = 10.13, 95% CI 1.33–77.00)), having mental distress (AOR = 2.69, 95% CI 1.02–7.06), playing online games (AOR = 2.40, 95% CI 1.38–4.18), current khat chewing (AOR = 3.34, 95% CI 1.14–9.83) and current alcohol use (AOR = 2.32, 95% CI 1.09–4.92) were associated with internet addiction.

**Conclusions:**

The current study documents a high prevalence of internet addiction among Wollo University students. Factors associated with internet addiction were spending more time, having mental distress, playing online games, current khat chewing, and current alcohol use. As internet addiction becomes an evident public health problem, carrying out public awareness campaigns may be a fruitful strategy to decrease its prevalence and effect. Besides to this, a collaborative work among stakeholders is important to develop other trendy, adaptive, and sustainable countermeasures.

## Background

Globally, more than three billion people use the internet daily with young people being the most common users [1]. In the field of medicine and healthcare, it helps in the practice of evidence-based medicine, research and learning, access to medical and online databases, handling patients in remote areas, and academic and recreational purposes [2, 3].

In terms of classical psychology and psychiatry, IA is a relatively new phenomenon. The literature uses interchangeable references such as “compulsive Internet use”, “problematic Internet use”, “pathological Internet use”, and “Internet addiction”. The Psychologist Mark Griffiths, one of the widely recognized authorities in the sphere of addictive behavior, is the author of the most frequently quoted definition: “Internet addiction is a non-chemical behavioral addiction, which involves human–machine (computer-Internet) interaction” [4, 5]. Internet addiction is a behavioural problem that has gained increasing scientific recognition in the last decade, with some researchers claiming it is a "21st Century epidemic"[6]. The psychopathologic symptoms of internet addiction includes Salience(the respondent most likely feels preoccupied with the Internet, hides the behaviour from others, and may display a loss of interest in other activities and/or relationships only to prefer more solitary time online), Excessive Use (the respondent engages in excessive online behaviour and compulsive usage, and is intermittently unable to control time online that he or she hides from others), Neglect Work (Job or school performance and productivity are most likely compromised due to the amount of time spent online), Anticipation(the respondent most likely thinks about being online when not at the computer and feels compelled to use the Internet when offline), Lack of Control(the respondent has trouble managing his or her online time, frequently stays online longer than intended, and others may complain about the amount of time he or she spends online) and Neglect Social Life (the respondent frequently forms new relationships with fellow online users and uses the Internet to establish social connections that may be missing in his or her life) [7–10].

Events during the adolescence period greatly influence a person's development and can determine their attitudes and behavior in later life [11]. The teenagers are often in conflict with authority and cultural and moral norms of society, certain developmental effects can trigger a series of defense mechanisms [12]. During adolescence, there is an increased risk of emotional crises, often accompanied by mood changes and periods of anxiety and depressive behavior, which some adolescents attempt to fight through withdrawal, avoidance of any extensive social contact, aggressive reactions, and addictive behaviour [13, 14]. Adolescents are exceptionally vulnerable and receptive during this period and can become drawn to the Internet as a form of release. Over time, this can lead to addiction [15].

Relaxed access and social networking are two of the several aspects of the Internet development of addictive behaviour [16]. Internet addiction is a newly emerged behavioral problem of adults which was reported after problem behavior theory was proposed [17]. Behavioral addictions operate on a modified principle of the classic addiction model [18–20]. Others have reported, that there is a tendency for individuals to be multiply ''addicted'' and to have overlapping addictions between common substances such as alcohol and cigarettes and ''addictions'' to activities such as internet use, gambling, exercising, and television [21]. A key factor to both models of substance and behavioral addictions is the concept of psychological dependence, in which no physiological exchange, such as ingestion of a substance, occurs [18, 22]. Internet addiction in puberty and young adults can negatively impact life satisfaction and engagement [23], which may negatively affect cognitive functioning [24], lead to poor academic performance [25, 26], and engagement in hazardous activities [27]. Internet addiction is also related to depression, somatization, and obsessive–compulsive disorder [28]. It has been found that paranoid ideation, hostility, anxiety, depression, interpersonal sensitivity, and obsessive–compulsive average scores are higher in people with high Internet Addiction scores than those without Internet addiction [29, 30].

College students are especially susceptible to developing a dependence on the Internet, more than most other segments of society. This can be qualified to numerous factors including the following: Availability of time; ease of use; the psychological and developmental characteristics of young adulthood; limited or no parental supervision; an expectation of Internet/computer use covertly if not, as some courses are Internet-dependent, from assignments and projects to link with peers and mentors; the Internet offering a way of escape from exam anxiety [31].

Studies have indicated that IA is associated with different factors. Socio-demographic factors such as age (having lower age) [32] and male gender [33–37]. Reason for internet use related factors such as making new friendships online [33], getting into relationships online [33], using the internet less for coursework/assignments [33], visiting pornographic sites [34] and playing online games [31, 34, 38]. Time related and other factors such as higher internet usage time [37, 39],continuous availability online [33, 35, 39] and mode of internet access [35]. Clinical and substance related factors such as insomnia [40], attention deficient disorder and hyperactivity symptoms [41], being sexual inactive [32], low self-esteem [40], failure in academic performance [32], smoking [41], and potential addictive personal habits of, drinking alcohol or coffee, and taking drugs [34]. Besides, mental illness like depression, anxiety and psychological distress [35–37, 39, 40] are associated with internet addiction. This could be based on the application of a general strain theory framework whereby negative emotions that are secondary to depression, anxiety, and psychological distress will be associated positively with internet addiction [42].

Internet Addiction is now becoming a serious mental health problem among Chinese adolescents. The researchers identified 10.6% to 13.6% of Chinese college students as Internet addicts [43, 44]. A study conducted among Taiwan college students reported that the prevalence of Internet Addiction was 15.3% [37].

The prevalence of Problematic Internet Use (PIU) was greater among university students. For instance, the prevalence was 36.9 to 81% in Malaysian medical students by using the internet addiction questionnaire and Internet Addiction Diagnostic Questionnaire study instrument with a cut-offs point of ≥ 43 and 31to 79 respectively [45, 46], 25.1% in American community university students by using the YIATstudy instrument with a cut-offs point of ≥ 40 [47], 40.7% in Iranian university students by utilizing the YIAT study instrument with a cut-offs point of ≥ 40 [48], 38.2–63.5% IA in Japanese university students as measured with the YIAT study instrument with a cut-offs point of ≥ 40 and ≥ 40 respectively [36, 49], 16.8% IA in Lebanon University students by utilizing the YIAT study instrument with a cut-offs point of ≥ 50 [40], 35.4% IA in Nepal undergraduate students as measured with the YIAT study instrument with a cut-offs point of ≥ 40 [32], 40% IA in Jordan University students by utilizing the YIAT study instrument with a cut-offs point of ≥ 50 [50],19.85% to 42.9% IA in various parts of India as measured with the YIAT study instrument with a cut-offs point of 31to79, ≥ 50 and ≥ 50 respectively [33, 35, 39], 12% IA to 34.7% (PIU) in Greek University students by utilizing the Problematic Internet Use Diagnostic Test study instrument with no stated cut-offs point [34], 1.6% IA in Turkey students by using the Young’s Internet Addiction Scale study instrument with a cut-offs point of 70–100 [41].

In general, the main reason why youths are at particular risk of internet addiction is that they spend most of their time on online gaming and social applications like online social networking such as Twitter, Facebook, and telegrams [51].

Even though developing countries shares for a large magnitude of internet addiction, indicating the public health impact of the problem in the region, much is not known about the occurrence rate of the problem in these regions in general and Ethiopia in particular. As a result, trustworthy assessments of internet addiction in university students in these circumstances are required for delivering a focused intervention geared towards addressing the associated factors.

Moreover, it will be a ground for the expansion of national and international plans, procedures, and policy. At last but not least, the findings from this study will provide significant implications for counsellors and policymakers to prevent students' Internet addiction. Hence, this a community university-based cross-sectional study aimed and assessed the prevalence and associated factors of internet addiction among Wollo university students.

## Research questions

The purpose of this study was to measure prevalence and associated factors of IA among undergraduate university students in Ethiopia. The specific research questions that guided the present study were:

1. What is the prevalence of IA among undergraduate university students in Ethiopia?
2. What are the associated factors of IA?

## Methods and materials

### Study area and period

The study was done at Wollo University, Dessie campus that is found in South Wollo Zone, Amhara Regional State which is 401 kms far from Addis Ababa, Northeastern Ethiopia. It had 5 colleges and 2 schools and a total of 62 departments. The number of regular students in 2018/2019 is 7248; among these 4009 are males and 3239 are females. The study was conducted from April 10 to May 10/ 2019. The sample size was determined using single population proportion formula, taking a 50% prevalence of Internet Addiction with the following assumption: 95% CI, 5% margin of error, 10% non-response rate, and a design effect of 1.5. So, the final sample size was 603.

### Sampling technique and procedure

A multistage cluster sampling technique was used to recruit study participants. In the first stage, by the use of the lottery method, two colleges (College of medicine and health sciences, and College of natural sciences, and one school (school of law)) were selected. In the second stage, 18 departments (9 from the college of medicine and health science, 8 from the college of natural science and 1 from the school of law) were selected. Students were selected proportionally from the given departments based on the number of students of a particular.

### Study design

A community university-based cross-sectional study was carried out to assess the prevalence and associated factors of Internet Addiction among undergraduate students at Wollo University, Amhara Region, Ethiopia.

### Inclusion and exclusion criteria

All generic regular undergraduate adult students whose ages were 18 years and above, and who were present at the time of data collection. Students who gave consent to the study were recruited. The study participants who are blind and severely ill were excluded from the study.

### Study instruments

Self-administered, well-structured, and organized English version questionnaire was disseminated to students, and data were collected from the individual student. The questionnaires comprised six parts. The first part consisted of socio-demographic details; a structured questionnaire was used to assess sociodemographic characteristics. The second part consists of Young’s Internet Addiction Test (YIAT); a structured, self-administered questionnaire was used to assess Internet Addiction. The YIAT [7] is the most commonly used measure of Internet Addiction among adults [52]. It includes 20 questions with a scoring of 1–5 for each question and a total maximum score of 100. Based on scoring subjects would be classified into normal users (0–30), mild (31–49), moderate (50–79), and severe (80–100) Internet Addiction groups. Mild Internet addiction, moderate Internet addiction, and severe Internet Addiction were considered as having an Internet Addiction [53–55]. YIAT-20 showed that it is more reliable in University students. The Cronbach α in the present study was 0.89. The third part time-associated factors; a self-report structured questionnaire was prepared from different kinds of literature to assess time-associated factors (such as Internet use experience in months and Internet use per day in hours). The fourth part reasons for internet use; a structured questionnaire was used to assess the reasons for internet use. The fifth part psychoactive substance use-associated factors; a self-report questionnaire was used to assess the current use of psychoactive substances (Khat, Cigarette, Alcohol, and Cannabis), and the last part mental health problem-associated factors and it was assessed by Kessler10 (K10). The K10 scale [56] is a simple measure of mental distress. The K10-item scale, which has been translated into Amharic and validated in Ethiopia [57], was used to measure mental distress (depressive, anxiety, and somatic symptoms). The internal consistency of the K10 psychological distress scale in the present study was checked with a reliability assessment and was found to be 0.86 [58–60]. Scores will range from 10 to 50. A score under 20 is likely to be well, a score of 20–24 is likely to have mild mental distress, a score of 25–29 is likely to have moderate mental distress and a score of 30 and over are likely to have severe mental distress. Study participants with a score of 20 or more points on the K10 Likert scale were considered as having mental distress [61].

### Data quality control

A structured self-administered questionnaire was developed in English and would be translated to Amharic language and again translated back into English to ensure consistency. Data collectors and supervisors would be trained for two days on the objective of the study, the content of the questionnaire, and the data collection procedure. Data would be pilot tested on 5% of the total sample size outside the study area and based on feedback obtained from the pilot test; the necessary modification would be done. During the study period, the collected data would be checked continuously daily for completeness by principal investigator and supervisor in the respective departments.

### Data processing and analysis

Quantitative data would be cleaned, coded, and entered into Epi-data 3.1 and exported to SPSS version 25 for analysis. Descriptive data would be presented by a table, graphs, charts, and means. Multicollinearity test was checked by using standard error and there was no correlation between independent variables. The association between independent variables and Internet Addiction would be made using a binary logistic regression model and all independent variables having *p* value ≤ 0.25 would be included in multiple logistic regression models. A *p* value less than 0.05 and Adjusted Odds Ratio (AOR) with 95% Confidence Interval (CI) not inclusive of one would be considered as statically significant and would be used to determine predictors of Internet Addiction in the final model. Hosmer–Lemeshow test was done to check model fitness and the model was fit.

## Results

### Socio-demographic characteristics of study participants

A total of 603 participants were involved with a response rate of 90.9% (n = 548). However, the rest 9.1% (n = 55) participants were excluded due to incomplete responses. The mean age of the respondents was 21.4 (SD 1.8) years, the minimum and maximum age of the participants was 18 years and 30 years respectively. More than half of, 291 (53.1%) of respondents were males. Many of the study participants had a practice of using the internet for more than twelve months, 321 (58.6%). About 501 (91.4%), 268 (48.9%), 433 (79%) were using the internet less than five hours per day, most common mode of internet access Wi-Fi, and log in and off occasionally during the day respectively. The study participants with current khat use, current cigarette smoking, current alcohol use, and current cannabis use were 19.0%, 11.3%, 25.4%; and 4.0% respectively. About 19.3% of the participants had mental distress (Table 1).

**Table 1:** Sociodemographic characteristics of study participants in Ethiopia, 2019 (n = 548)

Variables	Frequency	Percentage
*Age*		
Below mean	205	37.4
Above mean	343	62.6
*Sex*		
Male	291	53.1
Female	257	46.9
*Internet use experience (in months)*		
0 to 6 months	131	23.9
6 to 12 months	96	17.5
12 or more months	321	58.6
Internet use per day (in hours)		
Less than 5 h	501	91.4
Greater than or equal to 5 h	47	8.6
*The most common mode of Internet access*		
Wi-Fi	268	48.9
Broadband	16	2.9
Mobile internet	124	22.6
Data card	140	25.5
*Login status*		
Log in and off occasionally during the day	433	79.0
Permanently online	115	21.0
Current Khat chewer		
Yes	104	19.0
No	444	81.0
*Current cigarette smoker*		
Yes	62	11.3
No	486	88.7
*Current alcohol Drunker*		
Yes	139	25.4
No	409	74.6
*Current cannabis use*		
Yes	22	4.0
No	526	96.0
*Mental distress*		
Yes	106	19.3
No	442	80.7

### Prevalence of Internet addiction

**Fig. 1:**
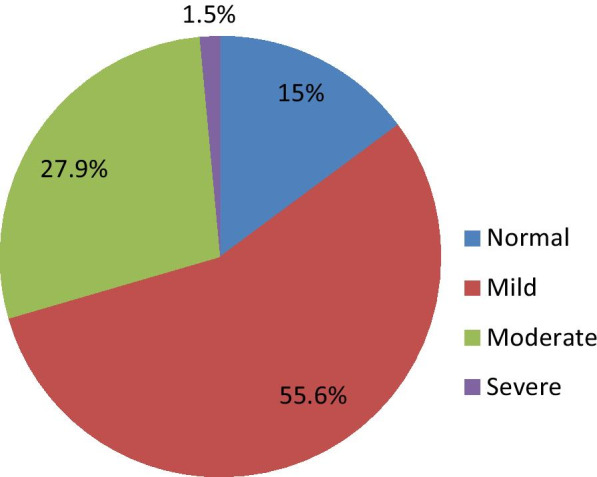
Internet Addiction by severity among undergraduate university students in Ethiopia, 2019 (n = 548)

The prevalence of IA was 466 (85%) of the 305(55.6%), 153(27.9%), 8(1.5%) mild, moderate, and severe Internet Addiction respectively. Nevertheless, the remaining 82 (15%) are free from Internet Addiction (Fig. 1). Participants who login permanently had a greater figure of IA than those who log in and off occasionally during the day (92.2% versus 83.1%). Those who used the internet for about six months had a greater prevalence of Internet Addiction than those who used greater than twelve months (91.6% versus 84.1%) (Table 2).

**Table 2:** Multivariable analysis of factors related to Internet Addiction among Undergraduate University Students in Ethiopia, 2019 (n = 548)

Variables	Internet addiction		COR (95% CI.)	AOR (95% CI.)	*p* Value
	Yes; N (%)	No; N (%)			
*Internet use experience (in months)*					
0 to 6 months	120(91.6%)	11(8.4%)	1	1	
6 to 12 months	76(79.2%)	20(20.8%)	0.35(0.16, 0.77)	0.42(0.18, 0.96)	0.040*
12 or more months	270(84.1%)	51(15.9%)	0.49(0.24, 0.96)	0.48(0.24, 0.99)	0.046*
*Internet use per day (in hours)*					
Less than 5 h	420(83.8%)	81(16.2%)	1	1	
Greater than or equal to 5 h	46(97.9%)	1(2.1%)	8.87(1.21, 65.25)	10.13(1.33, 77.00)	0.025*
*Most common mode of Internet access*					
Wi-Fi	230(85.8%)	38(14.2%)	0.84(0.45, 1.54)	0.84(0.44, 1.60)	0.596
Broadband	14(87.5%)	2(12.5%)	0.97(0.20, 4.63)	0.84(0.16, 4.35)	0.834
Mobile internet	99(79.8%)	25(20.2%)	0.55 (0.28, 1.07)	0.40(0.20, 0.83)	0.014*
Data card	123(87.9%)	17(12.1%)	1	1	
*Login status*					
Log in and off occasionally during the day	360(83.1%)	73(16.9%)	1	1	
Permanently online	106(92.2%)	9(7.8%)	2.39(1.16, 4.93)		
*Getting into relationships online*					
Yes	318(87.1%)	47(12.9%)	1.60 (0.99, 2.58)		
No	148(80.9%)	35(19.1%)	1	1	
*Playing mobile games*					
Yes	223(91.4%)	21(8.6%)	2.67(1.57, 4.52)	2.40(1.38, 4.18)	0.002*
No	243(79.9%)	61(20.1%)	1	1	
*Downloading music or videos*					
Yes	314(87.2%)	46(12.8%)	1.62(1.00, 2.61)		
No	152(80.9%)	36(19.1%)	1	1	
*Watching videos*					
Yes	281(88.6%)	36(11.4%)	1.94(1.21, 3.12)		
No	185(80.1%)	46(19.9%)	1	1	
*For retrieving sexual information*					
Yes	112(89.6%)	13(10.4%)	1.68(0.90, 3.15)		
No	354(83.7%)	69(16.3%)	1	1	
*Chat rooms*					
Yes	228(87.4%)	33(12.6%)	1.42(0.88, 2.29)		
No	238(82.9%)	49(17.1%)	1	1	
*Current Khat chewer*					
Yes	100(96.2%)	4(3.8%)	5.33(1.90, 14.91)	3.34(1.14, 9.83)	0.028*
No	366(82.4%)	78(17.6%)	1	1	
*Current cigarette smoker*					
Yes	61(98.4%)	1(1.6%)	12.20(1.67, 89.28)		
No	405(83.3%)	81(16.7%)	1	1	
*Current alcohol Drunker*					
Yes	129(92.8%)	10(7.2%)	2.76 (1.38, 5.51)	2.32(1.09, 4.92)	0.029*
No	337(82.4%)	72(17.6%)	1	1	
*Mental distress*					
Yes	101(95.3%)	5(4.7%)	4.26(1.68, 10.81)	2.69(1.02, 7.06)	0.045*
No	365(82.6%)	77(17.4%)	1	1	

^*^; Refers to a *p* value < 0.05 in the multivariable analysis and declared as statistically significant

1; Refers to a reference

### Reasons for internet use among Wollo University students

**Fig. 2:**
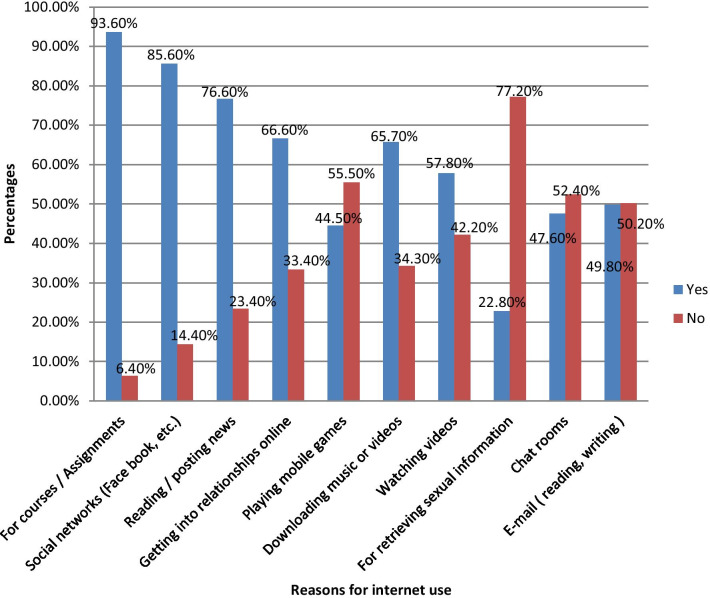
Reasons for internet use among undergraduate university students in Ethiopia, 2019 (n = 548)

The furthermost frequent reasons for internet use among Wollo University undergraduate students were using the internet for courses / assignments (93.6%), for social networks (Facebook, etc.) (85.6%), for reading / posting news (76.6%), for getting into relationships online (66.6%),for playing mobile games (44.5%), for downloading music or videos (65.7%), for watching videos (57.8%),for retrieving sexual information (22.8%), for chat rooms (47.6%) and for e-mail ( reading, writing) (49.8%) (Fig. 2).

### Factors associated with internet addiction in the univariate analysis

#### Time related factors

Duration of using the internet was associated with Internet Addiction i.e. students who used the internet for more than a year was 51% lower risks of having internet addiction than their counterparts (OR=0.49; CI 0.24–0.96). Respondents who were spending more time on the internet were more likely to develop Internet Addiction than their counterparts (OR=8.87; CI 1.21–65.25).

Mode of internet access was related to Internet Addiction i.e. those who used mobile internet were 45% lower risks of having Internet Addiction than those who used data cards (OR = 0.55; 95% CI 0.28–1.07).Participants who were permanently online were most likely to have Internet Addiction than those who were not (OR=2.39; 95% CI 1.16–4.93).

#### Reasons for internet use related factors

Study participants who played mobile games online were more likely to develop Internet Addiction than those who were not played mobile games (OR = 2.67; 95% CI 1.57–4.52). Those who downloaded music or videos were higher risks of having Internet Addiction than those who didn’t (OR = 1.62; 95% CI 1.00–2.61). Study participants who watched the video online were most likely to have Internet Addiction than those who didn’t watch (OR=1.94; 95% CI 1.21–3.12).

#### Psychoactive substance use related factors

Those who chewed khat currently were higher odds of having Internet Addiction than those who were not (OR = 5.33; 95% CI 1.90–14.91). Respondents who smoked cigarettes currently were more likely to have Internet Addiction than their counterparts (OR = 12.20; 95% CI 1.67–89.28).

Those who used alcohol currently were greater risks of having Internet Addiction than those who hadn't (OR = 2.76; 95% CI 1.38–5.51).

#### Mental health problem related factors

Study participants who had mental distress were four times more likely to develop Internet Addiction than those who didn't have mental distress (OR = 4.26; 95% CI 1.68–10.81) (Table 2).

### Factors associated with internet addiction in the multivariate analysis

In the final model, spending more time on the internet, having mental distress and playing online games were the factors associated with Internet Addiction. Moreover, current khat chewing and current alcohol use were the independent predictors for Internet Addiction. Using the internet for more than twelve months and using the internet by mobile internet were negatively associated with Internet Addiction (Table 2).

## Discussions

The present study aims to assess the prevalence and associated factors of Internet Addiction among undergraduate university students in Ethiopia. The prevalence of IA was 85% (n = 466). In the final model; spending more time on the internet, having mental distress and playing online games were the factors associated with Internet Addiction. Moreover, current khat chewing and current alcohol use were the independent predictors for Internet Addiction. Using the internet for more than twelve months and using the internet by mobile internet were negatively associated with Internet Addiction.

The prevalence of Internet Addiction in the present study was higher than the prevalence of Internet Addiction that was done in different universities such as three medical schools across three countries ( Croatia, India, and Nigeria) 49.7% [55], Malaysian 36.9% to 81% [45, 46], American community 25.1% [47], Iran 12.5 to 40.7% [48, 62, 63], Japan 38.2% to 63.5% [36, 49], Greek 12% to 30.1% [54, 64], Jordan 40% [50], Lebanon 16.8% [40], Nepal 35.4% [32] and in different parts of India 19.85% to 42.9% [33, 35, 39]. The discrepancy might be due to the cut-off point of YIAT-20, instrument difference, mental health policy, a cultural difference like time utilization, the difference in study participants such as in our study the participants were from two colleges and one school, and all participants were internet users, sample size and the time difference between the studies. The study in Malaysian University was conducted among medical students only and focusing on mild Internet Addiction and moderate Internet Addiction and not on severe Internet addiction.

In our study spending more time on the internet was 10 times more likely to develop Internet Addiction than those who are spending less time. The finding of this study is in line with similar studies done on college students in Taiwan and three medical schools across three countries (Croatia, India, and Nigeria) [37, 55]. The possible explanation for the association between Internet usage time and Internet Addiction is that it might be as much a symptom as it is a cause. However, this study design was cross-sectional and no causal relationship can be clarified, further studies ought to examine whether Internet usage time is an essential factor for determining Internet addiction.

Likewise, students who had mental distress were 2.7 times more likely to develop Internet Addiction as compared to their counterparts. Study findings in these areas showed that students who had mental distress were related to higher levels of Internet Addiction than students who hadn’t mental distress [35, 36, 39–41, 50]. This could be due to the Khantzian’s [65] self-medication hypothesis, indicating that mentally distressed university students might come to rely on the Internet as a method for coping with their mental distress. Hence, they will devote more and more time on the Internet and headway toward addiction if their mental distress symptoms are not cured [66].Students who had playing online games were 2.4 times higher to have Internet Addiction than their counterparts. A similar finding was also reported in Greek University and others [34, 38, 54, 67].

Furthermore, students who chewed khat currently were three times most likely to develop Internet Addiction than students who reported no current khat chewing which is in line with the study finding in Greek University students [34]. In this study, students who drank alcohol currently were 2.3 times most likely to have Internet Addiction as compared with students who didn’t drink alcohol. Other studies reported a similar finding [17, 34, 68, 69]. Probable reasons involve; based on the problem behavior theory, the problem behaviors (Internet Addiction and substance abuse) are inter-related.

Students who used the internet by mobile internet were 60% of lower risks of having Internet Addiction as compared to those students who used data cards. This might be due to inadequate finance to use the internet on mobile internet. So, the students may refrain from using the internet through mobile internet. Students who used the internet for more than 12 months were 52% less likely to have Internet Addiction than their counterparts. The current finding is not supported by other studies in the world. The present study has limitations such as alpha inflation from multiple testing and the analysis did not account for the complex sampling strategy in adjusting the standard errors.

## Conclusions

The current study documents a high prevalence of Internet Addiction among Wollo University students. The factors associated with Internet Addiction were spending more time on the internet, having mental distress, playing online games, current khat chewing, and current alcohol use. As internet addiction becomes an evident public health problem, carrying out public awareness campaigns on its severity and negative consequences of excruciating agonies may be a fruitful strategy to decrease its prevalence and effect. Campaign programs may aim at informing the adults on the phenomenon of internet addiction, knowing the possible risks and symptoms. Besides to this, a collaborative work among all stakeholders is important to develop other trendy, adaptive, ethical and sustainable countermeasures.

## Data Availability

The datasets supporting the conclusions of this article are not publicly available due to ethics regulations but may be available from the corresponding author upon reasonable request.

## Abbreviations

AOR: Adjusted odds ratio
CI: Confidence interval
COR: Crude odds ratio
IA: Internet addiction
K10: Kessler10
PIU: Internet addiction
SPSS: Statistical package for social science
YIAT: Young’s internet addiction test

## References

[1] Bremer J (2005). The internet and children: advantages and disadvantages. Child Adolesc Psychiat Clin.

[2] Swaminath G (2008). Internet addiction disorder: fact or fad? Nosing into nosology. Indian J Psychiat.

[3] Dargahi H, Razavi S. Internet addiction and its related factors: a study of an Iranian population. 2007.

[4] Tereshchenko S, Kasparov E (2019). Neurobiological risk factors for the development of internet addiction in adolescents. Behav Sci.

[5] Griffiths M (2000). Does internet and computer" addiction" exist? Some case study evidence. CyberPsychol Behav.

[6] Kuss D, Griffiths M (2014). Internet addiction in psychotherapy.

[7] Young KS (1998). Internet addiction: the emergence of a new clinical disorder. Cyberpsychol Behav.

[8] Morahan-Martin J, Schumacher P, editors. Incidence and correlates of pathological internet usage. Paper presented art the 105th annual convention of the American Psychological Association, Chicago, Illinois; 1997.

[9] Young KS, De Abreu CN. Internet addiction. A handbook and guide to evaluation. 2011.

[10] Scherer K (1997). College life online of college life and development. Healthy Unhealthy Internet Use J.

[11] Blos P (1966). On adolescence: psychoanalytic interpretation.

[12] Karacic S, Oreskovic S (2017). Internet addiction through the phase of adolescence: a questionnaire study. JMIR Mental Health.

[13] Achenbach TM, Becker A, Döpfner M, Heiervang E, Roessner V, Steinhausen HC (2008). Multicultural assessment of child and adolescent psychopathology with ASEBA and SDQ instruments: research findings, applications, and future directions. J Child Psychol Psychiat.

[14] Graovac M (2010). Adolescent u obitelji. Medicina Fluminensis: Medicina Fluminensis.

[15] ZboralskiACD K, OrzechowskaDEF A, TalarowskaDE M, DarmoszAB A, JaniakGF A, JaniakGF M, et al. The prevalence of computer and Internet addiction among pupils Rozpowszechnienie uzależnienia od komputera i Internetu. Postepy Hig Med Dosw(online). 2009;63:8–12. 19252459

[16] Kuss DJ, Griffiths MD (2011). Online social networking and addiction—a review of the psychological literature. Int J Environ Res Public Health.

[17] Ko CH, Yen J-Y, Yen CF, Chen CS, Weng CC, Chen CC (2008). The association between Internet addiction and problematic alcohol use in adolescents: the problem behavior model. Cyberpsychol Behav.

[18] Bradley BP (1990). Behavioural addictions: common features and treatment implications. Br J Addict.

[19] Holden C. 'Behavioral'addictions: do they exist?: American Association for the Advancement of Science; 2001. 10.1126/science.294.5544.980 11691967

[20] Miele GM, Tilly SM, First M, Frances A (1990). The definition of dependence and behavioural addictions. Br J Addict.

[21] Greenberg JL, Lewis SE, Dodd DK (1999). Overlapping addictions and self-esteem among college men and women. Addict Behav.

[22] Buckley RC (2015). Adventure thrills are addictive. Front Psychol.

[23] Shahnaz I, Karim A (2014). The impact of internet addiction on life satisfaction and life engagement in young adults. Univ J Psychol.

[24] Park M-H, Park E-J, Choi J, Chai S, Lee J-H, Lee C (2011). Preliminary study of Internet addiction and cognitive function in adolescents based on IQ tests. Psychiatry Res.

[25] Akhter N (2013). Relationship between internet addiction and academic performance among university undergraduates. Educ Res Rev.

[26] Usman N, Alavi M, Shafeq SM (2014). Relationship between internet addiction and academic performance among foreign undergraduate students. Proced Soc Behav Sci.

[27] Pfaff DW, Volkow ND. Neuroscience in the 21st century: from basic to clinical: Springer, Berlin, 2016.

[28] Alavi SS, Alaghemandan H, Maracy MR, Jannatifard F, Eslami M, Ferdosi M (2012). Impact of addiction to internet on a number of psychiatric symptoms in students of isfahan universities, iran, 2010. Int J Prevent Med.

[29] Xiuqin HHZ, Mengchen L, Jinan W, Ying Z, Ran T (2010). Mental health, personality, and parental rearing styles of adolescents with Internet addiction disorder. Cyberpsychol Behav Soc Netw.

[30] Alavi SSAH, Maracy MR, Jannatifard F, Eslami M, Ferdosi M (2012). Impact of addiction to internet on a number of psychiatric symptoms in students of Isfahan universities. Iran. Int J Prev Med.

[31] Kandell JJ (1998). Internet addiction on campus: The vulnerability of college students. Cyberpsychol Behav.

[32] Bhandari PM, Neupane D, Rijal S, Thapa K, Mishra SR, Poudyal AK (2017). Sleep quality, internet addiction and depressive symptoms among undergraduate students in Nepal. BMC Psychiat.

[33] Krishnamurthy S, Chetlapalli SK (2015). Internet addiction: prevalence and risk factors: A cross-sectional study among college students in Bengaluru, the Silicon Valley of India. Indian J Public Health.

[34] Frangos CC, Frangos CC, Sotiropoulos I (2011). Problematic internet use among Greek university students: an ordinal logistic regression with risk factors of negative psychological beliefs, pornographic sites, and online games. Cyberpsychol Behav Soc Netw.

[35] Gedam SR, Ghosh S, Modi L, Goyal A, Mansharamani H (2017). Study of internet addiction: Prevalence, pattern, and psychopathology among health professional undergraduates. Indian J Soc Psychiat.

[36] Kitazawa M, Yoshimura M, Murata M, Sato-Fujimoto Y, Hitokoto H, Mimura M (2018). Associations between problematic internet use and psychiatric symptoms among university students in Japan. Psychiat Clin Neurosci.

[37] Lin M-P, Ko H-C, Wu JY-W. Prevalence and psychosocial risk factors associated with Internet addiction in a nationally representative sample of college students in Taiwan. Cyberpsychol Behav Soc Netw. 2011;14(12):741–746. 10.1089/cyber.2010.0574 21651418

[38] Petry NM, O'Brien CP. Internet gaming disorder and the DSM‐5. 2013. 10.1111/add.12162 23668389

[39] Gupta A, Khan AM, Rajoura O, Srivastava S (2018). Internet addiction and its mental health correlates among undergraduate college students of a university in North India. J Family Med Primary Care.

[40] Younes F, Halawi G, Jabbour H, El Osta N, Karam L, Hajj A (2016). Internet addiction and relationships with insomnia, anxiety, depression, stress and self-esteem in university students: a cross-sectional designed study. PLoS ONE.

[41] Seyrek S, Cop E, Sinir H, Ugurlu M, Şenel S (2017). Factors associated with Internet addiction: Cross-sectional study of Turkish adolescents. Pediatr Int.

[42] Jun S, Choi E (2015). Academic stress and Internet addiction from general strain theory framework. Comput Hum Behav.

[43] Chou C, Hsiao M-C (2000). Internet addiction, usage, gratification, and pleasure experience: the Taiwan college students’ case. Comput Educ.

[44] Yang T, Yu L, Oliffe JL, Jiang S, Si Q (2017). Regional contextual determinants of internet addiction among college students: a representative nationwide study of China. Eur J Public Health.

[45] Ching SM, Awang H, Ramachandran V, Lim S, Sulaiman W, Foo Y (2017). Prevalence and factors associated with internet addiction among medical students-A cross-sectional study in Malaysia. Med J Malaysia.

[46] Haque M, Rahman NAA, Majumder MAA, Haque SZ, Kamal ZM, Islam Z (2016). Internet use and addiction among medical students of Universiti Sultan Zainal Abidin. Malaysia Psychol Res Behav Manag.

[47] Jelenchick LA, Becker T, Moreno MA (2012). Assessing the psychometric properties of the internet addiction test (IAT) in US college students. Psychiat Res.

[48] Bahrainian SA, Alizadeh KH, Raeisoon M, Gorji OH, Khazaee A (2014). Relationship of Internet addiction with self-esteem and depression in university students. J Prevent Med Hygiene.

[49] Tateno M, Teo AR, Shirasaka T, Tayama M, Watabe M, Kato TA (2016). Internet addiction and self-evaluated attention-deficit hyperactivity disorder traits among Japanese college students. Psychiatry Clin Neurosci.

[50] Al-Gamal E, Alzayyat A, Ahmad MM (2016). Prevalence of internet addiction and its association with psychological distress and coping strategies among university students in Jordan. Perspect Psychiat Care.

[51] Kuss DJ, Van Rooij AJ, Shorter GW, Griffiths MD, van de Mheen D (2013). Internet addiction in adolescents: prevalence and risk factors. Comput Hum Behav.

[52] Frangos CC, Frangos CC, Sotiropoulos I, editors. A meta-analysis of the reliabilty of young’s internet addiction test. Proceedings of the World Congress on Engineering; 2012: World Congress on Engineering London, United Kingdom.

[53] Young KS. Internet addiction test. Center for on-line addictions. 2009.

[54] Tsimtsiou Z, Haidich A-B, Spachos D, Kokkali S, Bamidis P, Dardavesis T (2015). Internet addiction in Greek medical students: an online survey. Acad Psychiatry.

[55] Balhara YPS, Gupta R, Atilola O, Knez R, Mohorović T, Gajdhar W (2015). Problematic internet use and its correlates among students from three medical schools across three countries. Acad Psychiatry.

[56] Kessler RC, Barker PR, Colpe LJ, Epstein JF, Gfroerer JC, Hiripi E (2003). Screening for serious mental illness in the general population. Arch Gen Psychiat.

[57] Kessler RC, Andrews G, Colpe LJ, Hiripi E, Mroczek DK, Normand S-L (2002). Short screening scales to monitor population prevalences and trends in non-specific psychological distress. Psychol Med.

[58] Tadegge AD (2008). The mental health consequences of intimate partner violence against women in Agaro Town, southwest Ethiopia. Trop Doct.

[59] Hanlon C, Medhin G, Selamu M, Breuer E, Worku B, Hailemariam M (2015). Validity of brief screening questionnaires to detect depression in primary care in Ethiopia. J Affect Disord.

[60] Fekadu A, Medhin G, Selamu M, Hailemariam M, Alem A, Giorgis TW (2014). Population level mental distress in rural Ethiopia. BMC Psychiat.

[61] Andrews G, Slade T (2001). Interpreting scores on the Kessler psychological distress scale (K10). Aust N Z J Public Health.

[62] Arbabisarjou A, Gorgich EAC, Barfroshan S, Ghoreishinia G (2016). The Association of internet addiction with academic achievement, emotional intelligence and strategies to prevention of them from student’s perspectives. Int J Hum Cult Stud (IJHCS)..

[63] Gorgich EAC, Moftakhar L, Barfroshan S, Arbabisarjou A. Evaluation of internet addiction and mental health among medical sciences students in the southeast of Iran. Shiraz E Med J. 2018;19(1).

[64] Frangos CCFC, Sotiropoulos I (2012). A meta-analysis of the reliability of young's internet addiction test. A meta-analysis of the reliability of young's internet addiction test. WCE..

[65] Khantzian EJ (1997). The self-medication hypothesis of substance use disorders: A reconsideration and recent applications. Harvard Rev Psychiat.

[66] Yen J-Y, Ko C-H, Yen C-F, Wu H-Y, Yang M-J (2007). The comorbid psychiatric symptoms of Internet addiction: attention deficit and hyperactivity disorder (ADHD), depression, social phobia, and hostility. J Adolesc Health.

[67] Association AP. Diagnostic and statistical manual of mental disorders (DSM-5®): American Psychiatric Pub; 2013. 10.1590/s2317-17822013000200017 24413388

[68] Sajeev Kumar P, Prasad N, Raj Z, Abraham A (2015). Internet addiction and substance use disorders in adolescent students-a cross sectional study. J Int Med Dent.

[69] Ko C-H, Yen J-Y, Chen C-C, Chen S-H, Wu K, Yen C-F (2006). Tridimensional personality of adolescents with internet addiction and substance use experience. Can J Psychiat.

